# Tungiasis presenting as a soft tissue oral lesion

**DOI:** 10.1186/1472-6831-14-112

**Published:** 2014-09-03

**Authors:** Elizabeth Sentongo, Henry Wabinga

**Affiliations:** 1 Department of Medical Microbiology, School of Biomedical Sciences, College of Health Sciences Makerere University, P. O. Box 7072, Kampala, Uganda; 2 Department of Pathology, School of Biomedical Sciences, College of Health Sciences Makerere University, P. O. Box 7072, Kampala, Uganda

**Keywords:** Lingual ectopic tungiasis, *Tunga penetrans*, Histopathology, Uganda

## Abstract

**Background:**

The sand flea *Tunga penetrans* usually infects the feet and affects primary school-age children and elderly persons in rural Uganda. Tungiasis occurs nationwide but disease outbreaks have been reported in the Busoga sub-Region of eastern Uganda, associated with poor sanitation and proximity between humans and domestic animals. Ectopic tungiasis, usually seen with extensive infection and at weight-bearing body surfaces often follows exposure in highly infested environments. For patients who present abroad treatment may be surgical excision or amputation.

**Case presentation:**

An adult female Musoga by tribe, resident in a Kampala City suburb presented at Mulago National Referral and Teaching Hospital’s Oral Surgery and Jaw Injuries Unit with a discoloured swollen tongue, facial cellulitis and submandibular lymphadenopathy. A swelling palpable in the body of her tongue was excised and sent for histology. Tungiasis of the tongue was diagnosed after microscopic examination of formalin-fixed paraffin-embedded Haematoxylin and Eosin-stained tissue sections.

**Conclusion:**

Lingual tungiasis is a rare diagnosis that was made on histological examination. Atypical presentation outside an endemic area predisposed the patient to partial glossectomy instead of the less invasive flea enucleation. Ectopic disease in a city-resident highlights the plight not only of visitors to infested areas but also of the communities and their domestic animals.

## Background

Tungiasis or sand flea disease caused by female *Tunga penetrans* (Linnaeus, 1758) and *Tunga trimamillata*[1] is an infection of the skin. The flea penetrates at periungual, interdigital or web sites, under toe nails or in grooves between toes and the ball of the foot, causing a mild inflammation. Infection can result in abscess formation, deformity and loss of digits. Tetanus is a potentially fatal complication of tungiasis [2,3]. Ectopic tungiasis has been described involving the hands and elbows in Tanzania [4], Cameroon [5] and Nigeria [6] and the palpebrae in the Democratic Republic of Congo [7]. Conjunctival [8], thigh, elbow and gluteal infections were reported in the Caribbean and South America [9,10] and growths over the ischial protuberances [11] and knee [12].

**Figure 1:**
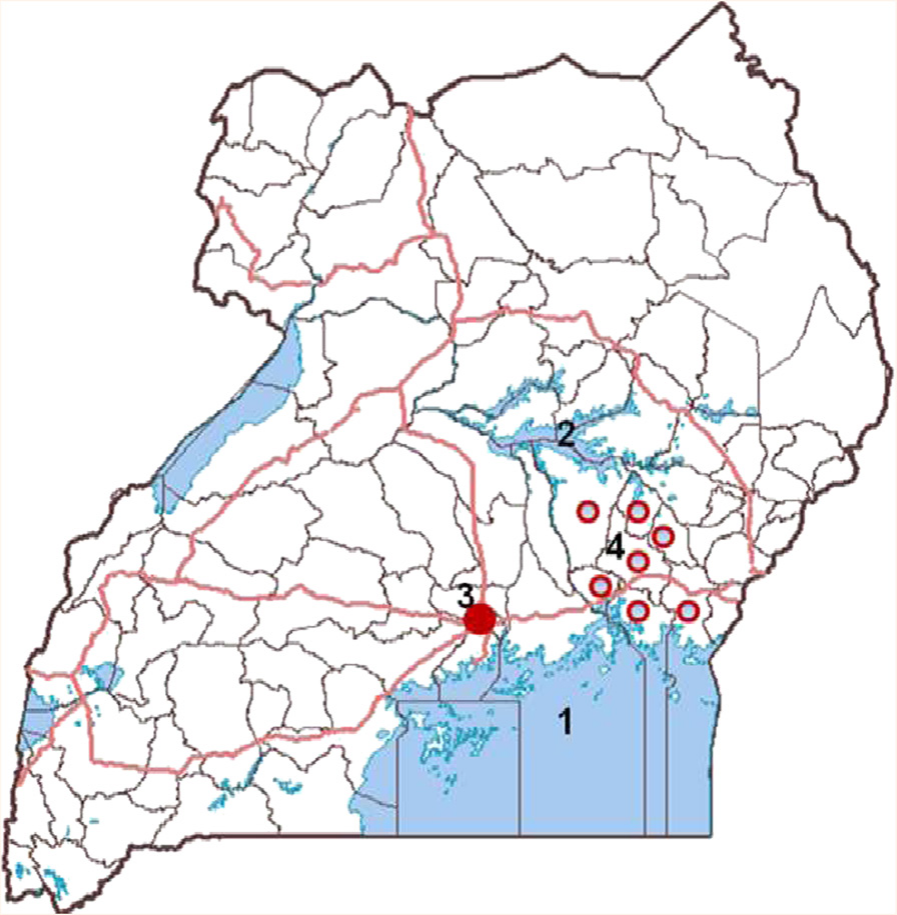
**A map showing the patient’s place of origin.** Lake Victoria **1**, Lake Kyoga **2**, Kampala City **3**, the Busoga sub-Region **4**. Graphical presentation used the HealthMapper application for the administrative divisions, then figures and numbers were affixed.

Heavy infection has been reported in eastern African communities with poor living conditions, high rat populations and in settlement camps [4,13-15]. Travellers, volunteers and immigrants present abroad with infection of the feet [16-18] and at times of the hands [19]. Many undergo surgical excision; where diagnosis is uncertain and disease is complicated a digit may be amputated [20]. Tungiasis particularly afflicts primary school-age children and elderly persons in rural Uganda. A survey of the Busoga sub-Region (Figure 1) following a disease outbreak in 2010 reported high prevalence, poor sanitation and proximity between humans and domestic animals [21]. National data on tungiasis is scanty but the disease is relatively confined even in highly affected areas. Here we report an unusual case of tungiasis, reflect on the conditions leading to infection and relate the options for individual treatment.

## Case presentation

A forty eight year old female Musoga by tribe, resident in Katwe suburb to the south of Kampala City (Figure 1) presented at Mulago National Referral and Teaching Hospital in the outpatients’ Oral Surgery and Jaw Injuries Unit on 25^th^ July 2011. She had had discolouration and swelling of the tongue for three weeks, difficulty in mastication and articulation and had taken antibiotics and analgesics. There was no history of trauma, dental manipulation, bleeding or swelling of other parts of the body or treatment for chronic disease. No social history had been recorded, her human immunodeficiency virus (HIV) serostatus was not known and the extremities were not inspected. Physical findings had noted a female in good general condition with swelling of the lower face and bilateral non-tender submandibular lymphadenopathy. The anterior part of the tongue was swollen, discoloured red and a firm tender lump measuring 2 × 4 millimetres was palpable. The mucosa, gingiva, palates, jaw and neck movements were normal.

### Diagnosis and management

**Figure 2:**
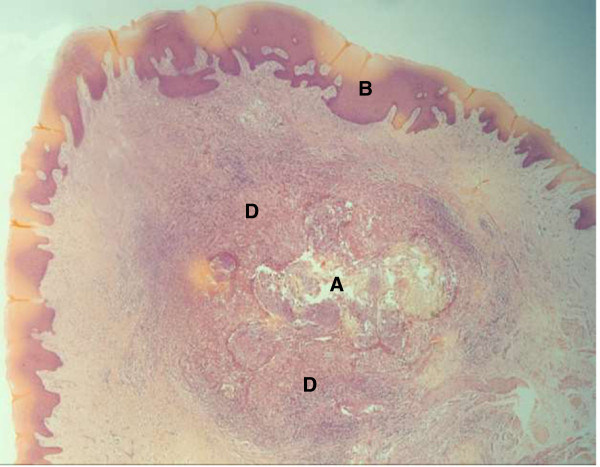
**A space-occupying lesion within tongue tissue (X2).** The lesion **A** is seen below the epithelium of the tongue **B**, surrounded by a granulomatous reaction **D**. Histology sections were prepared from surgically resected formalin-fixed paraffin-embedded tongue tissue and stained using Haematoxylin and Eosin.

**Figure 3:**
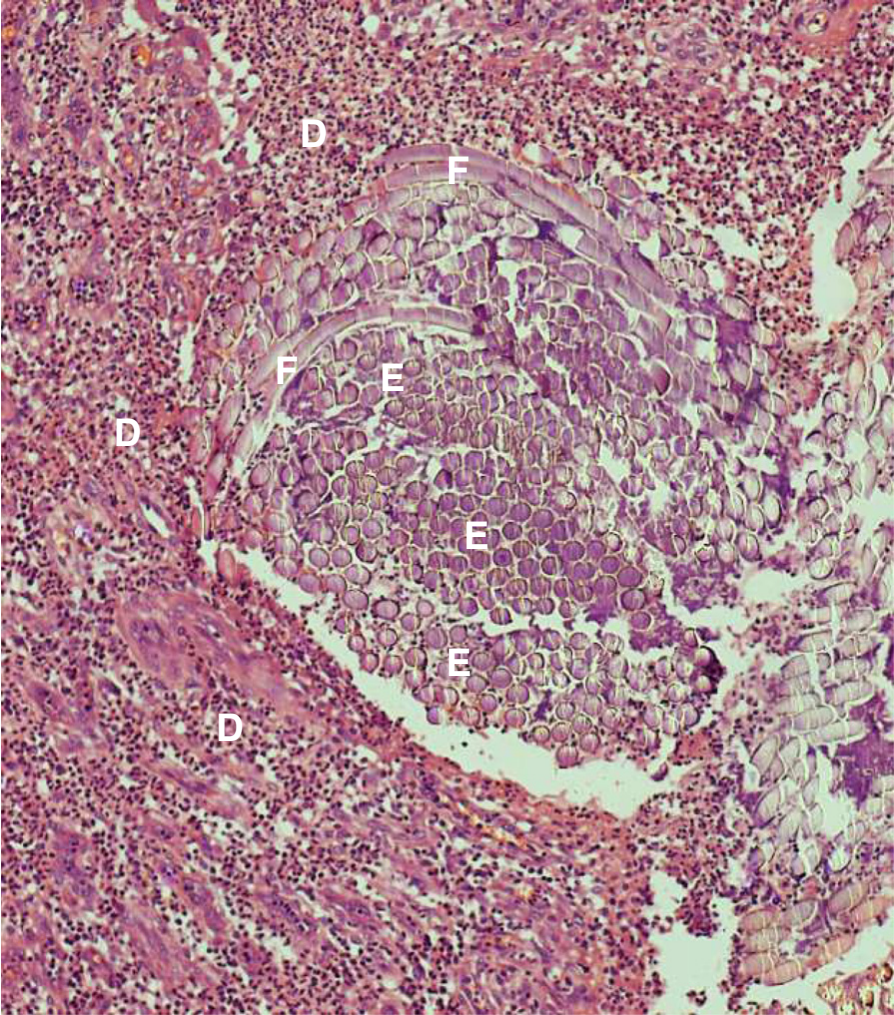
**The parasite’s exoskeleton enclosed compartments of ova (X10).** The exoskeleton **F** of the sand flea’s abdomen interceded between aggregations of ova **E** and the cellular infiltrate **D** predominantly of neutrophilic granulocytes, macrophages and giant cells.

**Figure 4:**
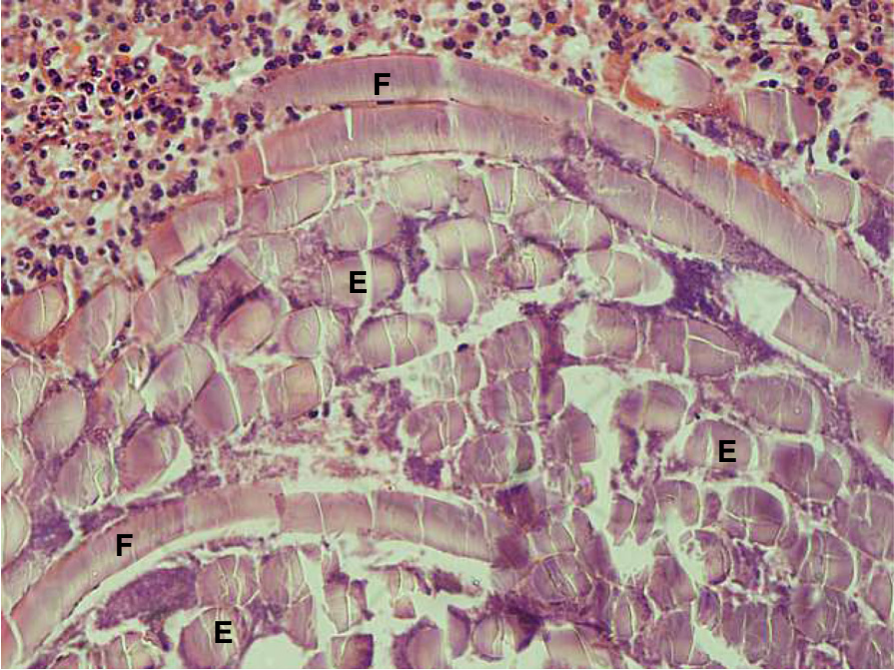
The ova E cracked and the flea’s rigid epicuticle F was inevitably disrupted and partly displaced by the tissue sectioning (X20).

Abnormal haemogram findings were a leucocytosis of 14.5 × 10^3^/μL and neutrophilia of 10.2 × 10^3^/μL (reference ranges 4–11 and 2–7 respectively), the bleeding indices were normal. With differential diagnoses of papilloma, granuloma, Kaposi sarcoma and cyst, the lump was excised and sent for histological examination. The patient was prescribed cetrimide-lidocaine hydrochloride mouth wash and gargle and a week later, the facial swelling and submandibular lymphadenopathy had subsided. There was no record of subsequent visits. Microscopy of formalin-fixed paraffin-embedded Haematoxylin and Eosin-stained tongue sections had shown a space-occupying lesion within skeletal muscle. This was surrounded by numerous neutrophils, macrophages and giant cells, some lymphocytes and occasional eosinophils. Several oval-shaped structures, some intact some cracked, aggregated in compartments that occupied most of the space. Discontinuous spiral-bound elongations at the periphery of the lesion represented the flea’s chitinous exoskeleton (Figures 2, 3 and 4). Based on the circular containment of numerous oval aggregations, elongated fractionated segments encapsulating the space and accumulation at the tissue-lesion interface of inflammatory cells characteristic of a foreign body reaction, tungiasis of the tongue was diagnosed.

## Discussion

The clinical findings were non-specific. A swollen hyperaemic tongue could have been reactive to a bite injury, friction irritation or allergen. A protuberance could be a papilloma, granuloma, malignancy or cyst and discolouration due to chemotherapy [22] or Kaposi sarcoma [23]. Community assessment of oral health conditions in ten Districts of Uganda yielded that apart from dental conditions, oral HIV lesions contributed 28.6%, oral cancer 10.3% and benign oral tumours 3.4% [24]. A study on Ugandans with AIDS-associated Kaposi sarcoma found that females more frequently presented with oral lesions [25]. In this case, histology found an embedded egg-bearing organism with features characteristic of tungiasis [17,18,26]. The diagnosis was further underscored by the patient’s tribe and place of origin, given the outbreaks and high infection rates in Busoga [21]. The circumstances of infection were intriguing. Exposure of the tongue would occur when the face was fairly close to the soil. This could be during home visits or cultural and social gatherings in the village when people sleep on the ground. The flea would access the tongue if nasal obstruction, habitual tongue protrusion or open-mouth posture kept the lips apart.

For a tungal lesion surgical excision was quite invasive. In the communities, the flea is enucleated preferably intact using a safety pin or thorn and the remnant ulcer doused with pepper extract, plant oil or petroleum product. The nuisance is thus removed and the parasite burden reduced. Extraction nonetheless requires caution in Busoga due to the high tetanus rates [27,28]. Alternative treatments are topical applications that suffocate the flea, which is then eliminated by the body’s repair mechanisms. These have been used in extensive infection where flea extraction would be overwhelming, highly traumatic or dangerous. In French Guiana 20% salicylated vaseline killed the parasites and facilitated their removal [10], in Brazil 0.8% ivermectin, 0.2% metrifonate, 5% thiabendazole and placebo lotions were compared [29] and an extract of coconut, jojoba and aloe vera cured and prevented new infections [30]. In highly infested areas, locally available products with a repellent effect are desirable; infections of the mouth may require a special oral paste.

## Conclusion

Tungiasis of the tongue in an adult resident of Kampala City is a rare diagnosis even for a native from Busoga. The atypical appearance and presentation outside the endemic area predisposed the patient to partial glossectomy rather than simple enucleation. Though invaluable, histological exclusion of the clinical diagnoses came late. This case, probably the first of lingual tungiasis, highlights the plight not only of visitors to infested areas but also of the communities and their domestic animals.

## Consent and permission

The patient gave written informed consent for treatment by the Oral Surgery and Jaw Injuries Unit of Mulago National Referral and Teaching Hospital. Informed consent for the publication of clinical findings and images was obtained; copies of the consent forms are available for review by the Editors of this journal. The Director General of Health Services, Uganda Ministry of Health gave permission to publish this case report and to use their Household Survey Report on Busoga.

## Abbreviations

AIDS: Acquired immune deficiency syndrome; HIV: Human immunodeficiency virus.

## Competing interests

The authors declare that they have no competing interests.

## Authors’ contributions

ES examined the tissue preparations for parasitic organisms, sought the patient’s details, obtained Ministry of Health Data and prepared the manuscript. HW examined the tissue preparations for neoplasms, malformations, abnormal growths, degenerations, irritants and non-parasitic organisms. Both authors read and approved the final manuscript.

## Authors’ information

1. ES’ qualifications: MBChB MPH Doktors der Medizin. Lecturer of Parasitology, Department of Medical Microbiology, School of Biomedical Sciences, Makerere University College of Health Sciences Kampala, Uganda

2. HW’s qualifications: MBChB MMed MD. Professor in the Department of Pathology, School of Biomedical Sciences, Makerere University College of Health Sciences Kampala, Uganda

The Medical Microbiology and Pathology Departments of Makerere University are housed within Mulago Hospital Complex and serve the Clinical and Pathology Laboratories of Mulago National Referral and Teaching Hospital. Patient information is kept on cards in the Outpatients’ Department. The histology report is kept in the Pathology Department of the School of Biomedical Sciences, Makerere University College of Health Sciences.

## Pre-publication history

The pre-publication history for this paper can be accessed here:

http://www.biomedcentral.com/1472-6831/14/112/prepub
